# Venom of Parasitoid, *Pteromalus puparum*, Suppresses Host, *Pieris rapae*, Immune Promotion by Decreasing Host C-Type Lectin Gene Expression

**DOI:** 10.1371/journal.pone.0026888

**Published:** 2011-10-26

**Authors:** Qi Fang, Fei Wang, John A. Gatehouse, Angharad M. R. Gatehouse, Xue-xin Chen, Cui Hu, Gong-yin Ye

**Affiliations:** 1 State Key Laboratory of Rice Biology & Key Laboratory of Molecular Biology of Crop Pathogens and Insects, Ministry of Agriculture, Institute of Insect Sciences, Zhejiang University, Hangzhou, China; 2 School of Biological and Biomedical Sciences, University of Durham, Durham, United Kingdom; 3 School of Biology, University of Newcastle-upon-Tyne, Newcastle-upon-Tyne, United Kingdom; Charité-University Medicine Berlin, Germany

## Abstract

**Background:**

Insect hosts have evolved immunity against invasion by parasitoids, and in co-evolutionary response parasitoids have also developed strategies to overcome host immune systems. The mechanisms through which parasitoid venoms disrupt the promotion of host immunity are still unclear. We report here a new mechanism evolved by parasitoid *Pteromalus puparum,* whose venom inhibited the promotion of immunity in its host *Pieris rapae* (cabbage white butterfly).

**Methodology/Principal Findings:**

A full-length cDNA encoding a C-type lectin (Pr-CTL) was isolated from *P*. *rapae*. Quantitative PCR and immunoblotting showed that injection of bacterial and inert beads induced expression of *Pr-CTL*, with peaks of mRNA and Pr-CTL protein levels at 4 and 8 h post beads challenge, respectively. In contrast, parasitoid venom suppressed *Pr-CTL* expression when co-injected with beads, in a time and dose-dependent manner. Immunolocalization and immunoblotting results showed that Pr-CTL was first detectable in vesicles present in cytoplasm of granulocytes in host hemolymph, and was then secreted from cells into circulatory fluid. Finally, the secreted Pr-CTL bound to cellular membranes of both granulocytes and plasmatocytes. Injection of double-stranded RNA specific for target gene decreased expression of *Pr-CTL*, and a few other host immune-related genes. Suppression of *Pr-CTL* expression also down-regulated antimicrobial and phenoloxidase activities, and reducing phagocytotic and encapsulation rates in host. The inhibitory effect of parasitoid venom on host encapsulation is consistent with its effect in suppressing *Pr-CTL* expression. Binding assay results showed that recombinant Pr-CTL directly attached to the surface of *P*. *puparum* egges. We infer that Pr-CTL may serve as an immune signalling co-effector, first binding to parasitoid eggs, regulating expression of a set of immune-related genes and promoting host immunity.

**Conclusions/Significance:**

*P*. *puparum* venom inhibits promotion of host immune responses by silencing expression of host C-type lectin gene *Pr-CTL*, whose expression affected transcription of other host immune-related genes.

## Introduction

Insects are subject to infection by a broad range of foreign invaders at different developmental stages [1], [2]. Like other arthropods, insects have an effective innate immune system to perform cellular and humoral responses. Microorganisms like bacteria and fungi, or parasitoid eggs, which pass through the cuticle into hemocoel of insect hosts, quickly trigger several immune responses [3]–[5]. The initial step of innate immune responses is to distinguish self from non-self by recognition of pathogen-associated molecular patterns present on the surface of microorganisms or other foreign tissue, mediated by pattern recognition receptors (PRRs) [6]–[8]. Successful recognition of “non-self” promotes multiple immune responses, such as phagocytosis, nodulation, encapsulation, syntheses of antimicrobial peptides and activation of the prophenoloxidase (proPO) system [4], [9]–[11].

The eggs and larvae of endoparasitoids develop in their host bodies, where they are subject to attack by host immune responses, including cellular and humoral responses, like encapsulation and melanization [12]. As a result of long-term co-evolution with their hosts, some hymenopteran parasitoids possess both maternal and embryonic active factors to positively suppress defences in their hosts [13], [14]. These factors include polydnaviruses (PDVs), virus-like particles contained in the ovary calyx fluid, proteins secreted from venom gland or ovaries, as well as teratocytes and their secreted proteins [13], [15]. Suppression of cellular immune responses is an important and common strategy used by parasitoids and some microbes to impair or circumvent host defence. For example, the hemocyte population and immunological functions such as spreading, phagocytosis and encapsulation of insect hosts are altered after parasitism or treatments with the immunosuppressive factors from parasitoids [5], [14], [16]–[22]. The humoral response of the host proPO activation cascade is also targeted and inhibited; for example, by a set of inhibitors present in the expression products of PDVs, or by secreted venom proteins of endoparasitoids [21], [23]–[26]. The immunosuppressive activity of parasitoids is highly specific, and dependent on specific interactions between molecules produced by the parasitoid and target molecules in the host.

The gregarious parasitoid, *Pteromalus puparum* (Hymenoptera: Pteromalidae) is a pupal parasitoid of *Pieris rapae* (cabbage white butterfly; Lepidoptera: Pieridae), a vegetable pest worldwide. This parasitoid injects venom, but not PDVs, into its host during oviposition. *P*. *puparum* and its host *P*. *rapae* comprise a model system for research into the influence of venom on host biology in system not dependent on PDVs [27], [28]. *P*. *puparum* venom causes alteration in the total number and morphology of host hemocytes [29], inhibits host cellular immune responses, including hemocyte spreading [30] and encapsulation [27], [31], and also decreases the phenoloxidase (PO) activity in host hemolymph [32]. However, the mechanisms which produce the suppressive effects of *P*. *puparum* venom on its host are not completely understood.

Our previous subtractive suppression hybridization and RT-PCR results showed that cDNA fragments encoding a C-type lectin of *P*. *rapae* (Pr-CTL) were differentially expressed in hemocytes from insects exposed to venom from *P*. *puparum* [32]. C-type lectins (CTLs) constitute the largest and most diverse family of animal lectins [33]. They function extracellularly and are secreted or membrane-bound [34]. CTLs are calcium-dependent carbohydrate-binding proteins that can bind terminal sugars on the surface of microorganisms [6]. In insects, CTLs play a key role in innate immune immunity, as PRRs, to promote cellular and humoral responses. Lepidopteran CTLs have been shown to participate in several immune responses, such as phagocytosis [35], nodule formation [36], [37], encapsulation and melanization [38]–[42].

This paper reports experiments designed to test the hypothesis that *P*. *puparum* venom inhibits promotion of host immune responses through suppression of expression of a gene encoding a C-type lectin. The outcomes of these experiments have given new insights into the mechanisms involved in successful parasitism.

## Results

### Molecular cloning and structural features of *Pr-CTL* gene

The cloning of a 3′-end fragment of a cDNA encoding C-type lectin, Pr-CTL, from a subtractive cDNA library prepared from hemocytes of *P*. *rapae* pupae has been described previously [32]. The full length cDNA of the *Pr-CTL* gene (GenBank accession number: JN133501) was obtained by rapid amplification of cDNA ends (RACE). The 1144 nt cDNA contained a 942 nt open reading frame (ORF) encoding an amino acid sequence of 313 residues, which included a predicted signal peptide of 19 residues (Fig. S1), giving a predicted mature protein with molecular weight 35.5 kDa and pI 5.1. The predicted sequence of Pr-CTL was analysed using the Pfam database, which showed the presence of two tandem carbohydrate recognition domains (CRDs; PF00059) spanning residues 41-151 and 184-302, respectively. This structural feature established that Pr-CTL belongs to C-type lectin family. The sequence contains 2 potential N-linked glycosylation sites, at residues 122-124 (NDT) and 271-273 (NAT). There were two tripeptides, “EPD” (117-119) and “EPN” (268-270) in first and second CRDs (Fig. S1). These two tripeptides are predicted to constitute the conserved mannose binding sites [43].

**Figure 1:**
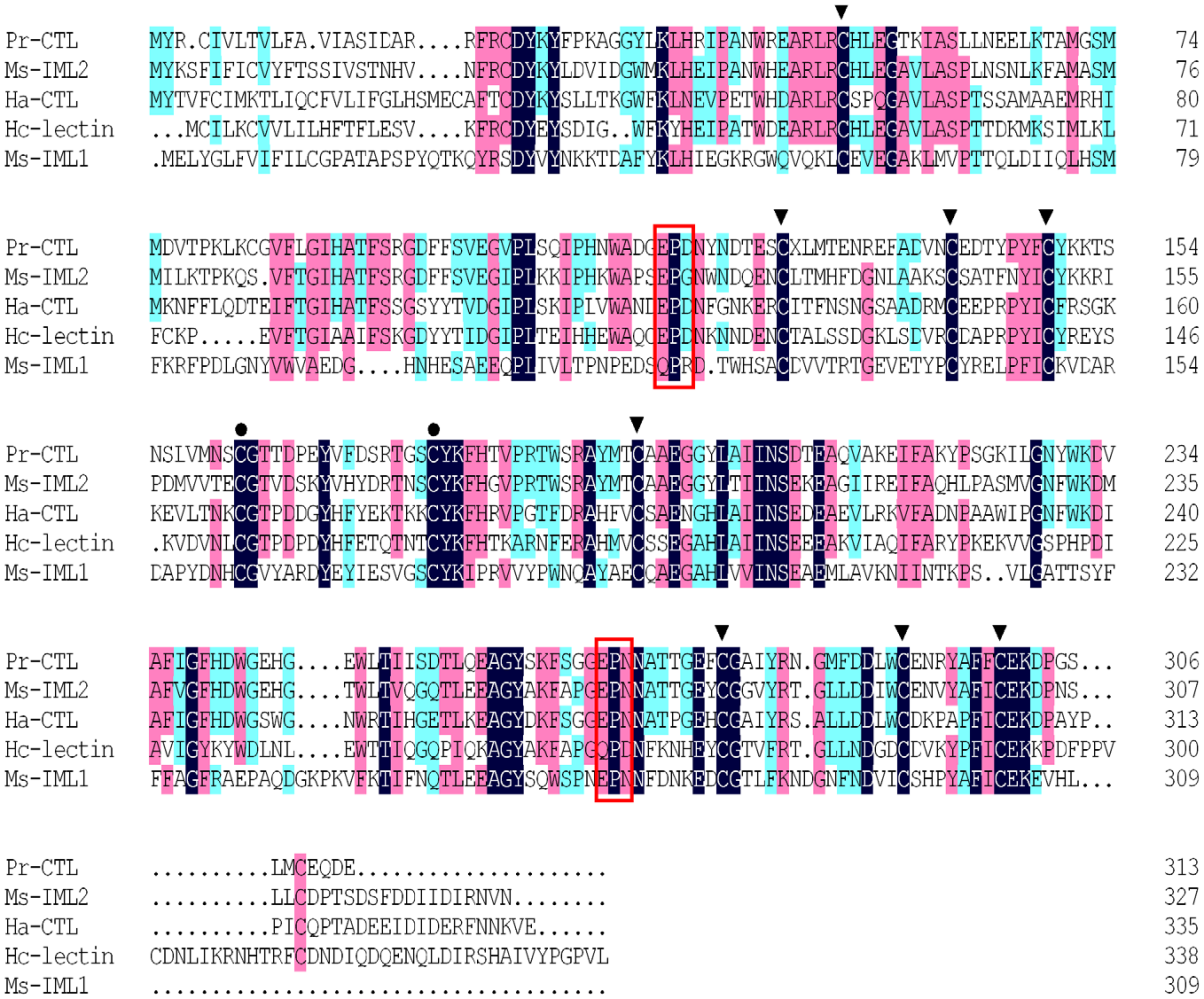
Multiple sequences comparison of insect C-type lectins. The sequences used are listed in the Table S1. Conserved amino acid residues are indicated by the same background color. The conserved cysteine (cys) residues that define C-type lectin short-form CRDs are marked with arrow head, whereas two extra cys residues in the long-form CRD are indicated by dots. Boxed residues indicate the potential carbohydrate specificity motifs.

A multiple sequence comparison and alignment with other insect C-type lectin sequences showed that Pr-CTL contains 10 conserved cysteine (C) residues (Fig. 1), of which the first four, C52, C127, C141 and C149, were present in the N-terminal CRD, and the remaining six, C162, C178, C195, C278, C292 and C300, were present in longer C-terminal CRD of Pr-CTL. The presence of two extra C residues in the longer C-terminal CRD compared to the shorter N-terminal CRD is a characteristic feature of the C-type lectin family. Similarity comparisons by BlastP showed that the amino acid sequence of Pr-CTL was most similar to immunlectin-2 of *Manduca sexta*, C-type lectin 21 of *Bombyx mori,* C-type lectin 2 of *Helicoverpa armigera* and the lectin of *Hyphantria cunea*, with amino acid residue identities of 60%, 57%, 49% and 43% respectively. A phylogenetic analysis of insect C-type lectins showed that Pr-CTL clustered with other lepidopteran CTLs, in a grouping distinct from *Drosophila melanogaster* C-type lectin, and from the out-group of *Caenorhabditis elegan*s (Fig. S2).

### Expression of recombinant Pr-CTL and antiserum preparation

The mature peptide of Pr-CTL was expressed as a 6xHis-tagged recombinant protein in *Escherichia coli*, using a PET vector system. Analysis of total cell proteins by SDS-polyacrylamide gel electrophoresis (SDS-PAGE, Fig. S3) showed that an expression of a polypeptide of approx. 36kDa was induced by Isopropyl β-D-1-thiogalactopyranoside (IPTG). Immunoblotting showed that this polypeptide contained a 6xHis tag. The polypeptide was purified under denaturing conditions by metal affinity chromatography. After separation by SDS-PAGE, the gel slice containing recombinant protein was used as an antigen to prepare polyclonal antiserum against Pr-CTL. The specificity and titer of the antiserum were assessed by enzyme-linked immunosorbent assay and immunoblotting results (data not shown), and were adequate for subsequent experiments.

### Effect of immune induction and inhibition on *Pr-CTL* gene expression

**Figure 2:**
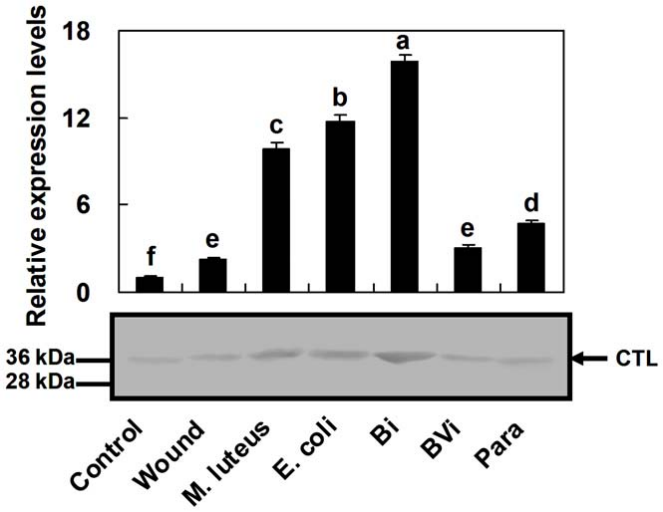
*Pr-CTL* transcripts levels (above) and Pr-CTL protein expression (below) following challenge treatments. Treatments included Control, PBS injection (Wound), *M*. *luteus* injection (M. luteus), *E*. *coli* injection (E. coli), bead injection (Bi), beads + venom injection (BVi), and parasitization (Para). For rq-rtPCR, each treatment was replicated 5 times. The values were represented as mean ± SE. SE bars annotated with the same letter are not significantly different (LSD test). The samples of total protein extracted from treated or control pupae were separated on 12% SDS-PAGE under reducing conditions and then immunoblotted, using anti-Pr-CTL antiserum as the first antibody. Molecular masses are indicated at the left of the blot. This blot represents the results of three biologically independent experiments.

Quantitative PCR was used to assay the expression level of the *Pr-CTL* gene in *P*. *rapae* pupae after immune challenge. The results of relative quantitative real-time PCR (rq-rtPCR, Fig. 2) showed that *Pr-CTL* gene transcript levels were sharply up-regulated by injections of bacteria (*Micrococcus luteus*, *E*. *coli* K12) and inert beads (Sephadex A-50), and that immune induction by beads caused the greatest increase in *Pr-CTL* mRNA levels. The transcript level induced by beads was 15.9 and 7.2 times higher than immunologically naive and Pringle's phosphate-buffered saline (PBS) injected *P*. *rapae* pupae, respectively, compared to increases of 11.8 and 5.4 times higher induced by *E*. *coli* and increases of 9.9 and 4.5 times higher induced by *M*. *luteus*.

In contrast, the level of *Pr-CTL* transcripts in pupae parasitized by *P*. *puparum* was only slightly higher than in PBS-injected pupae. Coinjection of venom + beads decreased the immune response caused by injection of beads, giving a level of transcript only 0.19 of that given by bead induction. Therefore, we infer that *P*. *puparum* venom could suppress the increase in expression level of the *Pr-CTL* gene, which occurred as part of the immune response. The levels of Pr-CTL protein detected by immunoblotting were consistent with the rq-rtPCR results (Fig. 2), in that levels were increased by immune challenge, but that increase did not occur when insects were parasitized, or were co-injected with venom + beads. These results suggest that alterations in the transcript levels of the *Pr-CTL* gene by immune stimulation and venom suppression correlated directly with the amounts of the encoded protein in the insect hemolymph.

### Time course of immune induction on *Pr-CTL* gene expression

**Figure 3:**
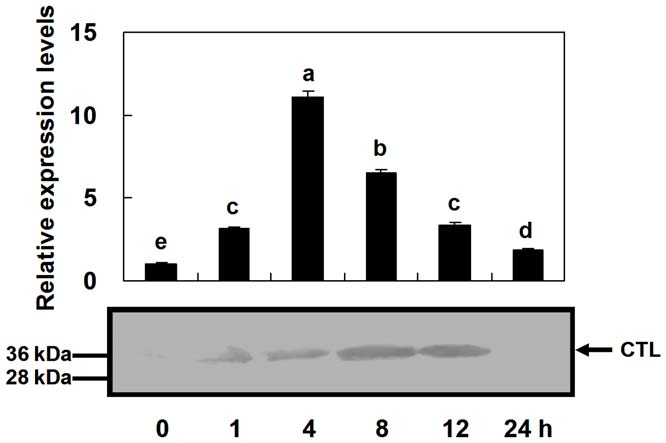
Time courses of host *Pr-CTL* transcript (above) and protein (below) expression following immune challenge. The details of rq-rtPCR and immunoblotting are presented in Figure 2. For rq-rtPCR, the values were represented as mean ± SE (n = 5). SE bars annotated with the same letter are not significantly different (LSD test). The arrow points to band representing the CTL protein.

Inert Sephadex beads were used to induce an immune response in *P*. *rapae* pupae, mimicking eggs of *P*. *puparum*, and the mRNA expression level of the *Pr-CTL* gene was measured at varying times after immune induction. The rq-rtPCR results (Fig. 3) suggested *Pr-CTL* gene expression increased from 0 to 4 h after immune challenge, with a peak of transcript accumulation at 4 h, 11.0 times higher than 0 h. From 4 to 24 h post treatment, the transcript level of the *Pr-CTL* gene fell to only 1.8 times higher than 0 h. The amounts of Pr-CTL protein detected by immunoblotting (Fig. 3) were consistent with the rq-rtPCR results, but there was a lag between transcript accumulation and protein accumulation. The maximum content of Pr-CTL in total protein extracted from pupae appeared at 8 h post beads injection, 4 h later than the appearance of the transcript peak. The protein expression levels of 8 and 12 h were similar, but the Pr-CTL level had decreased significantly by 24 h, and Pr-CTL was not able to be detected by immunoblotting.

### Tissue distribution of *Pr-CTL* gene expression

**Figure 4:**
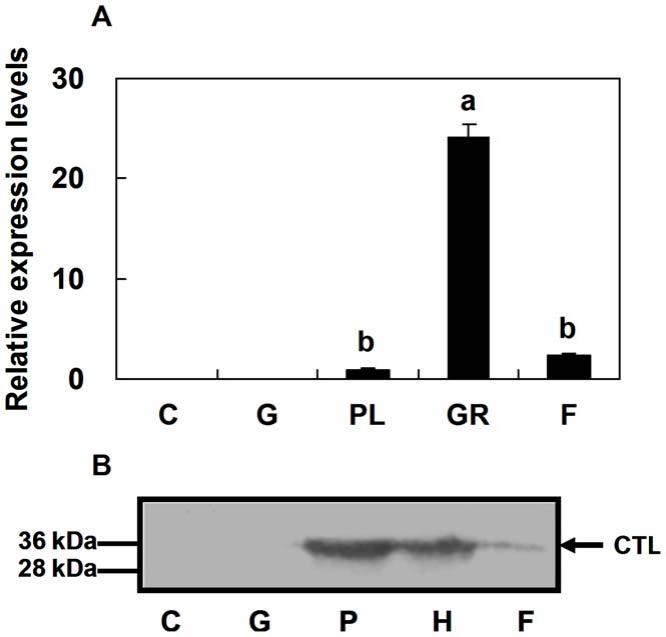
*Pr-CTL* transcript and protein expression levels in the indicated tissues following immune challenge. Panels A and B represent mRNA and protein expression profiles, respectively. The analyzed samples include cuticle (C), gut (G), plasmatocytes (PL, only for rq-rtPCR), granulocytes (GR), fat body (F), and cell-free plasma (P, only for immunoblotting). For rq-rtPCR, each histogram bar represents the mean ± SE (n = 5) of transcript levels. SE bars annotated with the same letter are not significantly different (LSD test). The protein samples were analyzed by 12% SDS-PAGE under reducing conditions and immunoblotting using anti-Pr-CTL antiserum. Molecular masses are indicated to the left of the blot and the arrow points to the band representing the CTL protein. This blot represents the results of three biologically independent experiments.

The mRNA expression level of the *Pr-CTL* gene after immune challenge by bead injection was assayed in different tissues and hemocytes. The rq-rtPCR results (Fig. 4) illustrated that the *Pr-CTL* gene expression was only detectable in plasmatocytes, granulocytes and fat body, and not in cuticle and gut. Gene expression was highest in granulocyte cells in the hemolymph, and was also present in fat body and plasmatocytes. The levels in granulocytes and fat body were 24.1 and 2.7 times higher than plasmatocytes, respectively. These results are consistent with Pr-CTL being involved in immunity, since it is expressed by hemocytes and fat body, which are the main effectors for insect innate immunity. Immunoblotting for Pr-CTL protein gave results consistent with the mRNA assays, with Pr-CTL being detected in total protein from both hemocytes and fat body, not in gut and cuticle. The Pr-CTL protein level in hemocytes was higher than fat body. Pr-CTL was also present in the plasma of *P*. *rapae,* based on the immunoblotting results, at a higher level even than hemocytes. This result is consistent with secretion of Pr-CTL into the plasma as a soluble protein after expression in host hemocytes and fat body.

### Localization of endogenous Pr-CTL in *P*. *rapae* pupal hemocytes

**Figure 5:**
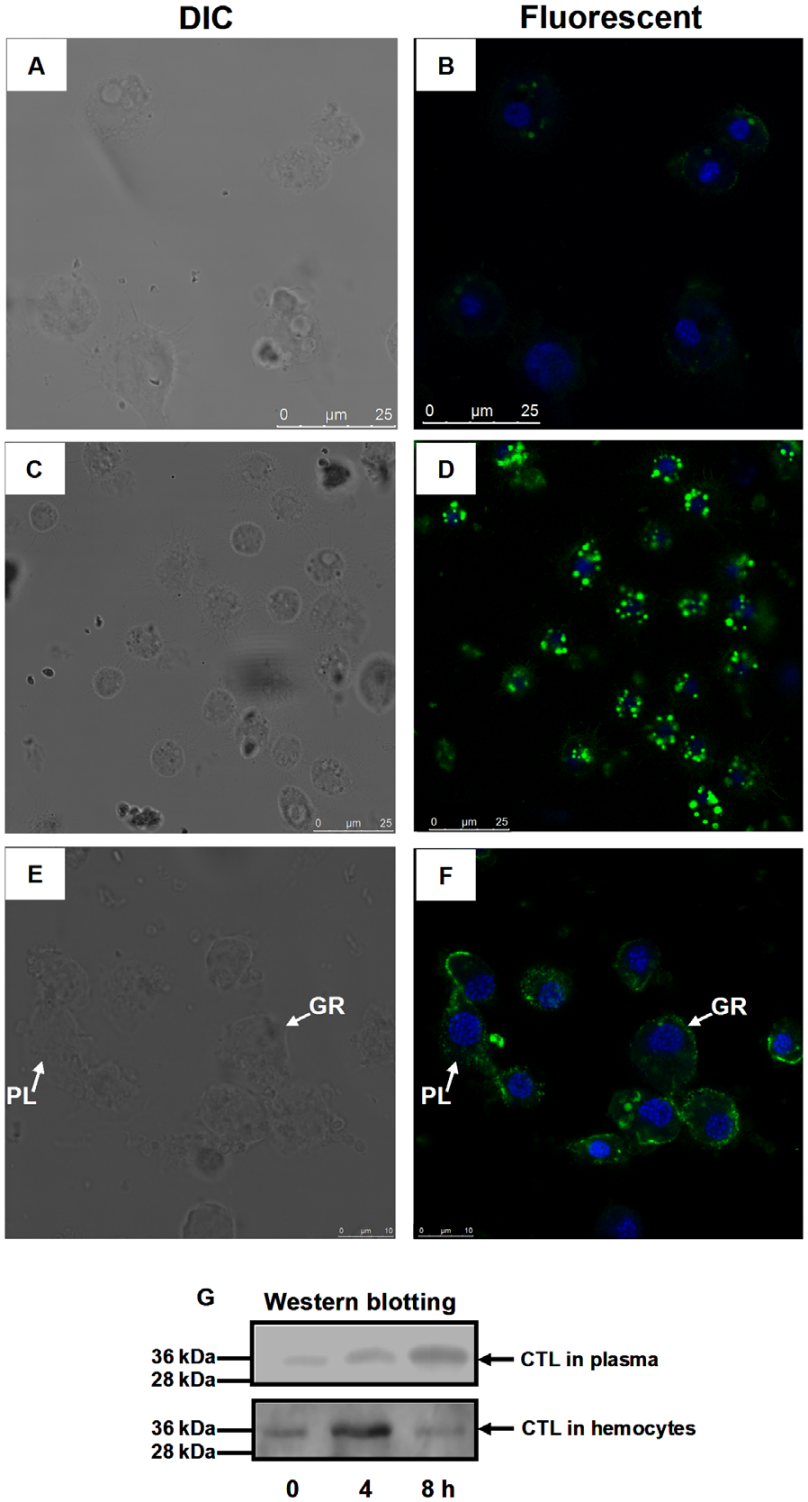
Time courses of the *Pr-CTL* gene expression in hemocytes and plasma following immune challenge. The confocal observations are performed at 0 (panels A and B), 4 (C and D), and 8 h (E and F) post immune challenge, respectively. Granulocytes (GR) and plasmatocytes (PL) are indicated in both panel E (DIC) and F (Fluorescent). In panels A to D, bar  =  25 µm, and in panels E and F, bar  =  10 µm. Panel G shows the expression profiles of *Pr-CTL* in plasma (above) and hemocytes (below) at different sampling time post immune challenge. Molecular masses are indicated to the left of the blot and the arrow points to the band representing the CTL protein.

Since the *Pr-CTL* gene was mainly expressed in host hemocytes, an immunolocalization assay was performed to demonstrate the presence of Pr-CTL protein in hemocytes, and the dynamics of its synthesis and transport after immune induction. As shown in Fig. 5, at 0 h post induction, Pr-CTL could only be detected in the cellular membrane and cytoplasm of some hemocytes at very low levels. Pr-CTL in cytoplasm was aggregated to form small granules (Fig. 5 A, B).

At 4 h post induction, most hemocytes showed prominent granulation. In these granulocytes, the level of Pr-CTL was greatly increased. Pr-CTL was mainly present in the cytoplasm, and was localized to the granules, rather than distributed, throughout the cytoplasm (Fig. 5 C, D). At 8 h post induction, the prominent granules in hemocytes were decreased. Pr-CTL was mainly detected in cell membrane of hemocytes, and the granules formed by aggregated Pr-CTL almost disappeared (Fig. 5 E, F). In parallel, the content of Pr-CTL in host plasma and hemocytes from 0 to 8 h post immune induction was assayed using immunoblotting. The results (Fig. 5G) showed that the content of Pr-CTL was gradually increased from 0 to 8 h in plasma, and was maximal at 8 h. In contrast the level of Pr-CTL in hemocytes showed a peak at 4h post-immune induction, with the content at 0 and 8 h relatively low. According to the results above, we infer that Pr-CTL is mainly expressed in granulocytes, and could be released into plasma as a soluble protein crossing their membranes. It was surprising that Pr-CTL could be observed in both granulocyte and plasmatocyte membranes at 8 h. It implies that Pr-CTL may bind to the surface of both two types of host hemocytes.

**Figure 6:**
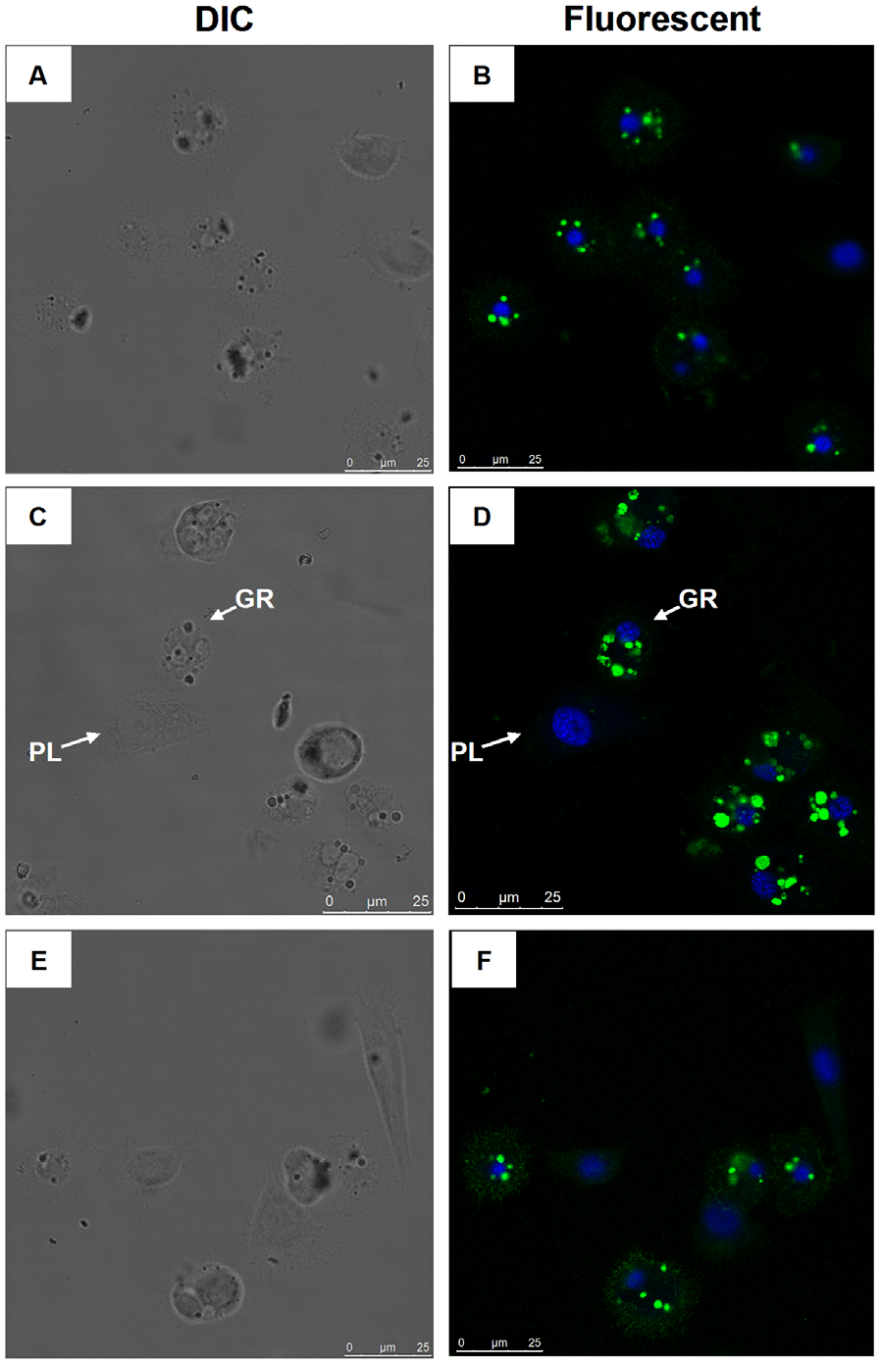
The influence of venom on *Pr-CTL* gene expression in host hemocytes. The confocal observations were carried out at 4 h after treatments. Panels A and B represent hrmocytes collected from the pupae only injected with PBS (control). Panels C and D show hemocytes collected from the pupae injected with 5-10 beads. Panels E and F show hemocytes collected from the pupae co-injected with beads plus venom. Granulocytes (GR) and plasmatocytes (PL) are indicated in panel C (DIC) and D (Fluorescent). Bar  =  25 µm.

Moreover, we selected 4 h post immune induction as the time point to investigate whether the treatment with *P*. *puparum* venom would change the expression profile of *Pr-CTL*. The results showed that post beads challenge (Fig. 6 C, D), the expression of *Pr-CTL* was enhanced, compared to that of the granulocytes only injected with PBS (Fig. 6 A, B). After beads + venom treatment, the expression of *Pr-CTL* was not induced to the same level as by beads alone (Fig. 6 E, F), and its level was similar to the PBS treatment. This result suggests that venom could down-regulate the expression of *Pr-CTL* in granulocytes. We also found the expression level of *Pr-CTL* in plasmatocytes was consistently lower than in granulocytes when compared under different treatments, in agreement with the rq-rtPCR results reported above.

### Functional analysis of *Pr-CTL* by RNA interference

**Figure 7:**
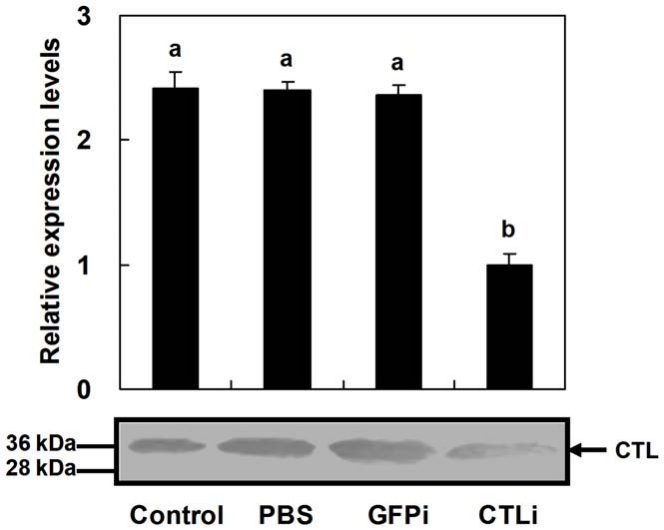
dsRNA treatments suppressed *Pr-CTL* transcript and protein expression. Control pupae were punctured to control for wound effects (Control), PBS to control for the dsRNA vehicle (PBS) and dsRNA targeted to the *EGFP* gene (GFPi) to control for adventitious effects of non-specific dsRNA. The suppressing effects of dsRNA were recorded by rq-rtPCR (above) and immunoblotting (below). For rq-rtPCR, each treatment was replicated 5 times, and the histogram bars representmean ± SE. SE bars annotated with the same letter are not significantly different (LSD test). For immunoblotting, the shown blot represents the results of three biologically independent experiments.

**Figure 8:**
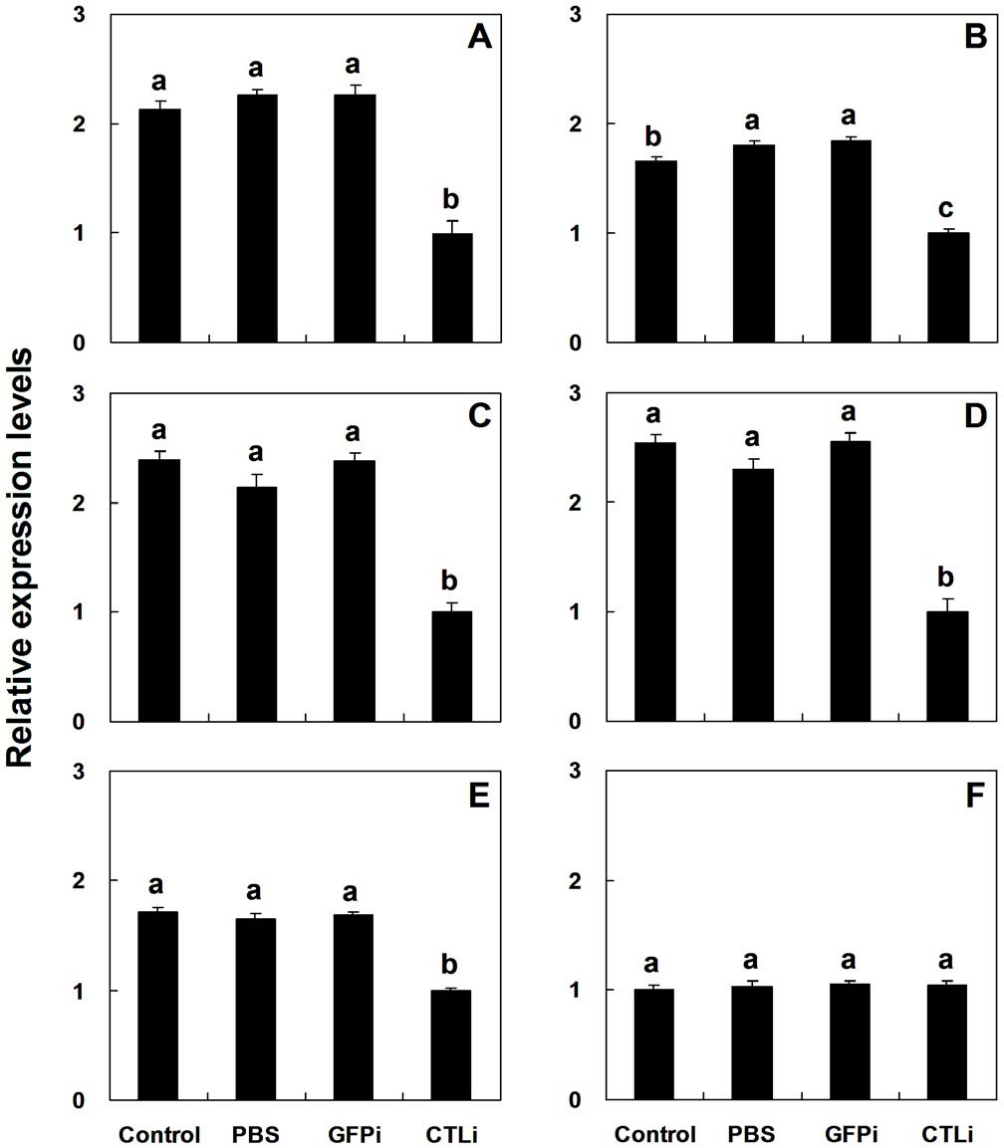
The influence of dsRNA on the transcript and protein expression levels of immune-related genes. Tested genes included *Pr-cecA* (A), *Pr-lys* (B), *Pr-PAP1* (C), *Pr-PAP3* (D), and *Pr-SR* (E) and the control gene *Pr-GST* (F). The details of treatments and controls are described in Fig. 7. The histogram bars represent the mean ± SE (n = 5) transcript levels. SE bars annotated with the same letter are not significantly different (LSD test).

Injection of double-strand RNA (dsRNA) targeting the *Pr-CTL* gene into *P*. *rapae* pupae caused significant down-regulation of gene expression post immune challenge, shown by rq-rtPCR (Fig. 7). Compared to controls, the mRNA expression level of *Pr-CTL* was down-regulated to approx. 35% of control level. Immunoblotting results suggested RNAi also could decrease the level of Pr-CTL protein (Fig. 7). The transcript levels of 5 immune-related genes of the host *P*. *rapae*, *Pr-cecA, Pr-lys, Pr-PAP1, Pr-PAP3* and *Pr-SR*, which are involved in antimicrobial activity, proPO activation, and phagocytosis were also decreased post-RNAi treatment targetting the *Pr-CTL* gene, with mRNA levels reduced to 40-60% of controls (Fig. 8 A, B, C, D, E). The largest effect was seen for *Pr-PAP3*. These results suggest that decreasing the expression level of the *Pr-CTL* gene affects expression of other immune-related genes. However, the transcript level of the host control gene, glutathione s-transferase gene (*Pr-GST*), which may play roles in detoxification of xenobiotics by *P*. *rapae,* was not significantly affected after RNAi treatment (Fig. 8 F). These data show that Pr-CTL specifically regulates host immune-related genes.

**Figure 9:**
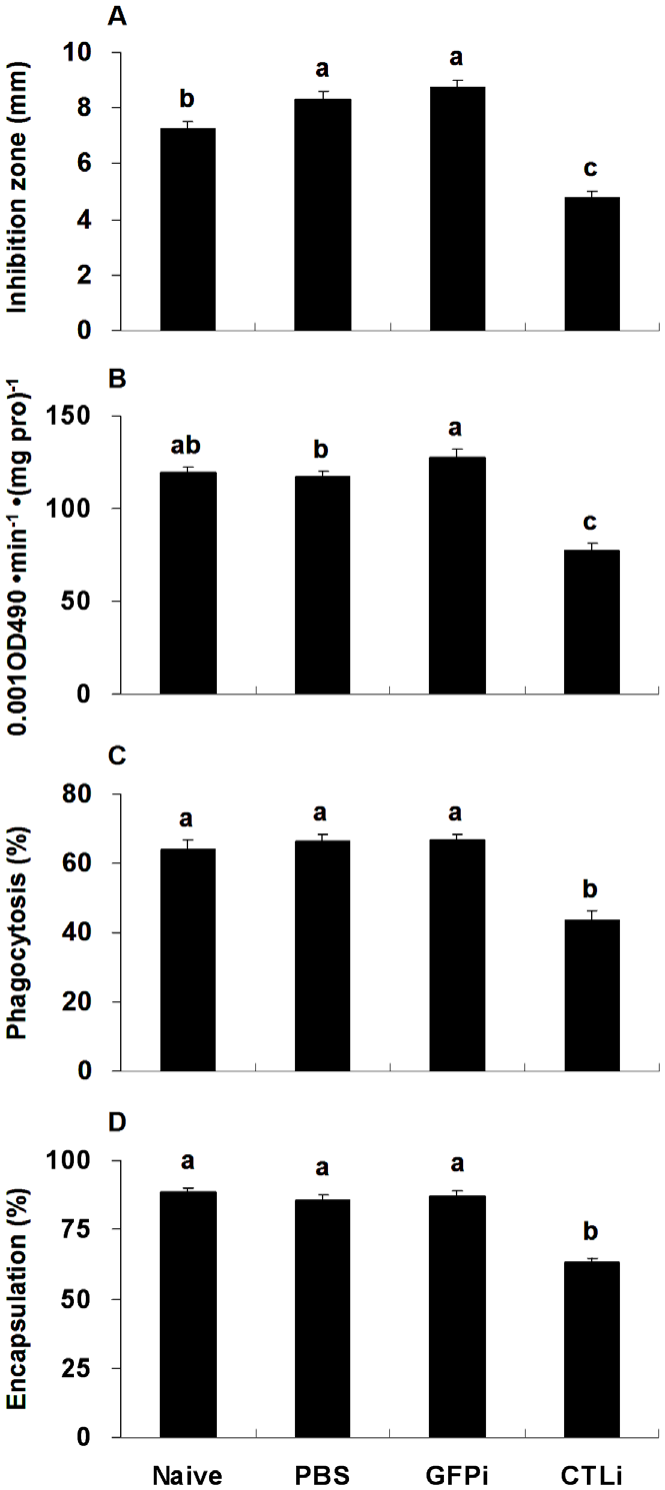
The influence of dsRNA on host immune responses. The responses assayed include hemolymph antimicrobial (A) and PO (B) activities, as well as hemocytes phagocytosis (C) and encapsulation (D) capabilities. All experiments were performed *in vitro*. The details for all treatment and controls setting are described in Fig. 7. Each histogram bar represents mean ± SE (n = 5). SE bars annotated with the same letter are not significantly different (LSD test).

To confirm that down-regulation of the *Pr-CTL* gene could affect immune responses at the phenotypic level, the effect of dsRNA on antimicrobial, PO, phagocytosis and encapsulation activities were assayed. In all cases, injection of dsRNA targetting the *Pr-CTL* gene decreased the immune responses (Fig. 9). Decreases in these responses ranged from nearly 50% (antimicrobial acitivity, PO activity) to approx. 30% (phagocytosis, encapsulation). All of these effects were statistically significant (for antimicrobial activity, *F*_3, 16_ = 40.86,*P* = 0.0001; for PO activity, *F*_3, 16_ = 42.31,*P* = 0.0001; for phagocytosis, *F*_3, 16_ = 30.80,*P* = 0.0001; for encapsulation, *F*_3, 16_ = 10.47,*P* = 0.0005) after RNAi treatment to *Pr-CTL* gene. These results suggest that the *Pr-CTL* gene not only contributes to cellular but also to humoral immune responses of the host.

### Time course and dose effect of venom inhibition on *Pr-CTL* gene expression

**Figure 10:**
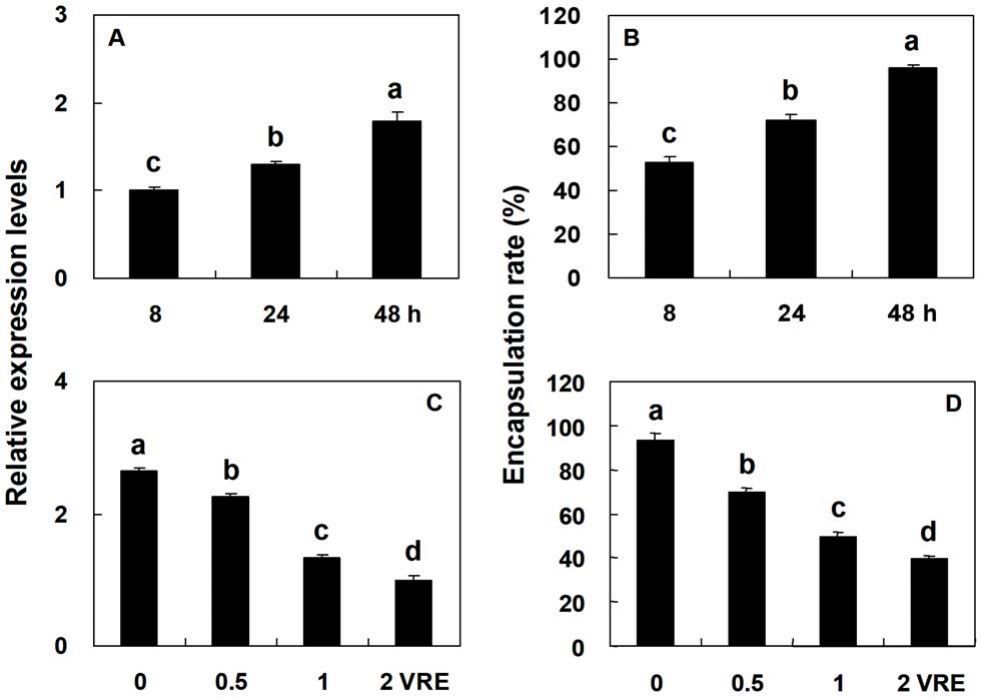
Influence of time course and venom dose on *Pr-CTL* transcripts level and encapsulation capacity. Panels A and B represent the influence of time course, while panels C and D represent of venom dose. *Pr-CTL* transcript levels were determined by rq-rtPCR, and encapsulation rates were determined *in vitro*. For time course experiments, 2 VREs were used for each time point. For dose effect, both the transcripts level and *in vitro* encapsulation rate were determined at selected times post treatment (incubations at room temperature). Each histogram bar represents the mean ± SE, n = 5. SE bars annotated with the same letter are not significantly different (LSD test).

The effects of *P*. *puparum* venom expression on *Pr-CTL* gene expression decreased after the initial effect; transcript levels of *Pr-CTL* remained low during the first 8 h following the bead + venom treatments, but increased about 2-fold over the next 40h (Fig. 10, panel A). The influence of venom treatments on immune responses was consistent with the levels of *Pr-CTL* mRNA detected; the encapsulation rate for beads increased approx. 2-fold over the 8-48h post-injection period (Fig. 10 B). A similar correlation between the effects of venom dose on *Pr-CTL* expression and inhibition of encapsulation was observed (Fig. 10 C, D). These results provide additional evidence for Pr-CTL regulation of immune gene expression, and immune functions.

### Localization of recombinant Pr-CTL on the surface of *P*. *puparum* egg

**Figure 11:**
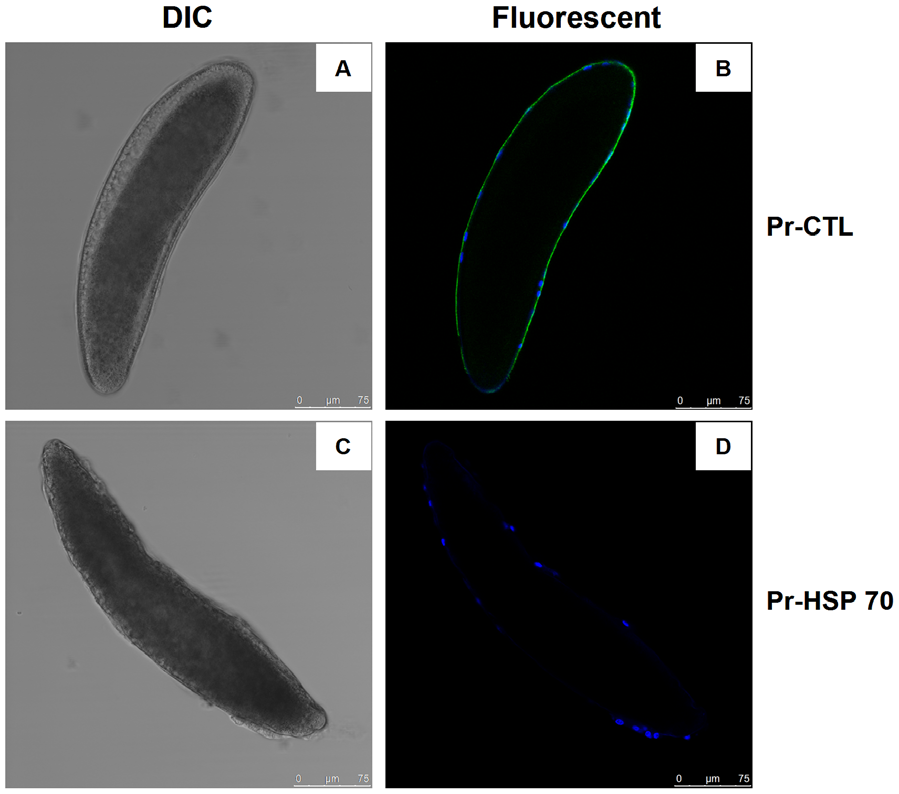
Binding of recombinant Pr-CTL to the surface of *P*. *puparum* egg. The confocal observations are performed at 1 h post parasitoid eggs treated with recombinant Pr-CTL (A and B) and Pr-HSP 70 (Control, C and D), respectively. The binding of recombinant proteins to the cells on the surface of eggs is represented by green pattern, and the nuclei of the cells are represented by blue color. In each panel, bar  =  75 µm. Panels B and D are fluorescent views, while panels A and C are DIC views of the eggs.

Recombinant Pr-CTL and *P*. *rapae* heat shock protein Pr-HSP 70 (as a control host protein, recombinantly expressed by our lab previously), both containing a 6 × His-tag at their N-terminuses were incubated with *P*. *puparum* eggs, and binding of recombinant proteins to wasp eggs was detected by monoclonal anti His-tag antiserum. Recombinant Pr-CTL, but not Pr-HSP 70, was detected on the surface of the *P*. *puparum* egg (Fig. 11), suggesting that binding of Pr-CTL to surface molecules on wasp eggs is an initial step in promoting one or more immune-related signalling pathways in the host.

## Discussion

The main response in lepidopteran larvae and pupae to parasitism by endoparasitoids is the formation of a melanized capsule, composed of multiple layers of host hemocytes around the parasitoid eggs. The attached hemocytes and the melanin formed may lead the death of the encapsulated parasitoid eggs [12]. Previous studies verified that venom plays an important role in the immunosuppression of hosts that are parasitized by *P*. *puparum* [27], [30]–[32]. After infection by *P*. *puparum* or treatment with venom, host hemocytes lose the capacity to adhere to the surfaces of foreign bodies, which blocks encapsulation and allows the offspring of *P*. *puparum* to develop in host hemocoel [29]. Humoral responses like hemolymph melanization are also inhibited by *P*. *puparum* parasitism or treatment with its venom [27], [32]. Both cellular and humoral responses depend on non-self recognition performed by host hemocytes and the related immune signalling transduction pathway, triggered by some receptors or effectors in host immune system [6]–[8]. Therefore, results shown in this paper indicate that the C-type lectin Pr-CTL may play a role as a primary immune signalling co-effector to promote immune responses in the host *P*. *rapae*, since its down-regulation suppresses expression of a wide range of immune-related genes, which were included in both cellular and humoral responses.

The C-type lectin Pr-CTL from hemocytes of *P*. *rapae* contains two CRDs, an organization that is similar to that of lipopolysaccharide biding protein from *B*. *mori* [36], and to CTLs from other lepidopteran species, including *M*. *sexta* [40], [44], [45], *H*. *cunea* [46], [47], *Lonomia obliqua* and *H*. *armigera* [48]. These insect C-type lectins form a distinct group, differing from most animal CTLs that contain a single CRD (including most mammalian CTLs). The insect lectins with tandem CRDs may have increased binding affinity to carbohydrates on the surface of pathogens compared to their mammalian equivalents which only have a single CRD [49]. Our primary results of carbohydrates binding assay show that recombinant Pr-CTL possesses the calcium-dependent agglutination of different kind of carbohydrates, such as mannose, galactose and peptidoglycan (data not shown).

We evaluated effects of different immune stimulation on the expression on *Pr-CTL* gene by rq-rtPCR and immunoblotting methods. The results indicate that gram-negative *E*. *coli*, gram-positive *M*. *luteus* and Sephadex beads strongly induced *Pr-CTL* gene expression, similar to results for other insect species. For example, in *M*. *sexta*, the transcripts levels of some immune lectin (IML) genes are up-regulated by *E*. *coli* challenge, and the IMLs contents in total hemolymph protein are also increased by treatment with bacteria and fungi, respectively [40], [41], [44]. Additionally, the mRNA and protein expression levels of the CTL gene isolated from the hemocytes of *H*. *armigera* are both significantly induced by *Bacillus thuringiensis*, *Staphylococcus aureus*, *Pichia pastoris* and nuclear polyhedrosis virus, but not by *E*. *coli* [48]. In *Anopheles gambiae*, the mRNA expression levels of two CTLs, CLT4 and CTLMA2, are increased by gram-negative bacteria, at 12 h post treatments [33]. The results reported here show that *Pr-CTL* gene expression in host hemocytes is constitutive, but the transcript level is low prior to immune challenge, and Pr-CTL protein could not be detected in total protein extracts of whole pupae by immunoblotting. Both mRNA and protein expression levels of *Pr-CTL* peak after immune induction, which suggests that turnover is relatively rapid, and these levels of the protein in non-challenged insects are likely to be low.

The results of the expression profile associated with tissue distribution indicate that *Pr-CTL* gene expression mainly occurs in hemocytes of *P*. *rapae*, especially in granulocytes, with limited expression in fat body. This is similar to the expression profile of a CTL gene from *H*. *armigera* [48], and the CTLs BmLEL-1 and BmLEL-3 from *B*. *mori* [50]. However, it is different from different types of CTLs identified in other insects, which are only expressed in fat body [36], [40], [41], [44], [45], [50], [51]. *Pr-CTL* is first expressed by hemocytes and fat body, mainly by hemocytes, and then may be secreted into plasma. This conclusion is consistent with results obtained for the CTL from *H*. *armigera* [48]. Similar to other insects, *P*. *rapae* may possess multiple CTL genes, with differing expression profiles; the results obtained for *Pr-CTL* may only apply to this member of the gene family.

The detection of Pr-CTL protein in hemocytes by immunolocalization prior to immune induction verifies again that *Pr-CTL* gene expression in host hemocytes is constitutive. We infer that the constitutively produced Pr-CTL is required for the primary step of non-self pattern recognition. At 4 h post immune induction, the content of Pr-CTL in granulocytes is increased, and the protein aggregates to form the granules, as shown in Fig. 5D. This is different from the immunolocalization results for IML-3 isolated from *M*. *sexta* [45]. However, IML-3 is also located in cytoplasm of hemocytes. The subcellular expression profiles of *Pr-CTL* and IML-3 are different. Although IML-3 is also observed in the cytoplasm of hemocytes of *M*. *sexta*, and appears as large vesicles, scattered IML-3 is present in cytoplasm too. In *P*. *rapae* hemocytes, we only detect aggregated Pr-CTL and not free Pr-CTL in the cytoplasm. At 8 h after beads induction, we find that Pr-CTL is located mainly in the cellular membrane, and the Pr-CTL accumulated as vesicles disappears. This pattern is similar to the localization of endogenous C-type lectins DL2 and DL3 in *Drosophila* hemocytes, where binding to the cell membranes is also observed [6].

CTLs are important in innate immune responses of mammals, because they can recognize pathogens and directly function as effectors to neutralize or clear them [49], [52]–[55]. Insects may use molecules such as CTLs to recognize infectious pathogens and simulate protective defenses, because they lack antibodies or clonal selection of lymphocytes [44]. Therefore, CTLs are essential and important components of insect immune system and may function as recognition proteins binding to the surface of invaders [6], [7], [44]. Down-regulation of *Pr-CTL* expression by RNAi causes reductions in the transcripts levels of other immune-related genes of *P*. *rapae*, including cecropin, lysozyme, PAPs and scavenger receptor genes, and also significant decreases in the cellular and humoral responses of *P*. *rapae* against immune challenge, including antimicrobial activity, melanization of hemolymph, phagocytosis and encapsulation of the host. It is suggested that Pr-CTL may serve as a co-effector in host immune signalling pathway to regulate expression of a set of immune-related genes and then to promote many responses of *P*. *rapae* immunity. If the expression level of *Pr-CTL* gene is down-regulated, many host immune responses will be suppressed.

In other insect species, CTLs also take part in the important immune pathways. For instances, IMLs of *M*. *sexta* activated proPO cascades [41], [44], enhanced the cellular encapsulation and melanization [38], [40], and protected larvae of *M*. *sexta* against bacterial infection [56]. In *B*. *mori*, lipopolysaccharide binding protein (C-type lectin) might aid aggregation of hemocytes to form nodules [36]. Two CTLs cooperate to defend *A*. *gambiae* against gram-negative bacteria [33]. All this evidence is consistent with the results reported for Pr-CTL in this paper. More importantly, our results show that Pr-CTL expression affects expression of other immune-related genes in the host.

Following identification of the parasitoid egg surface as foreign by recognition molecules, proteolytic cascades are activated and the hemocyte encapsulation response is initiated [13]. Non-self recognition is the first step of innate immunity and then immune signalling pathways are triggered. Wasp parasitism involves immunosuppressive factors which regulate the promotion of host immunity, including recognition or immune signalling transduction. In agreement with the data presented in this paper, other recent results suggest that immunosuppressive factors of parasitoids influence the expression of the host CTL genes, or competitively substitute the CTL binding of the host to effectively disrupt the pattern recognition. For example, the polydnavirus from *Hyposoter didymator* inhibits the CTL gene expression of its host, *Spdoptera frugiperda* [57], and the CTL homolog expressed by bracovirus from *Cotesia plutellae,* whose amino acid sequence has high identity with IML-2 of *M*. *sexta,* suppressed the cellular responses of its host, *Plutella xylostella* [16], [58], by masking hemocyte-binding sites on the parasitoid eggs. Our results show that the transcripts and protein levels of *Pr-CTL* in *P*. *rapae* are significantly triggered by beads injection treatment, but the addition of venom in parasitization and beads + venom treatments respectively suppresses the levels of both transcript and protein compared to treatments where venom is absent. We conclude that *P*. *puparum* venom suppresses the expression of the host *Pr-CTL* gene, which is otherwise increased by immune challenge. The correlation between timecourse and dose dependency of Pr-CTL gene expression after venom injection, and the immune response observed, and the specific binding of recombinant Pr-CTL to the surface of parasitoid eggs support the conclusion that Pr-CTL is a causal factor in the immune response. It may serve as an important binding protein targetted to parasitoid eggs and as a co-effector to take part in the host immune signalling transduction. Injection of *P*. *puparum* venom inhibits the promotion of host immune responses by decreasing the host Pr-CTL expression.

Taken together, the results of this paper support the hypothesis that suppression of expression of a gene encoding a C-type lectin is one mechanism that allows *P*. *puparum* to successfully parasitize its host, *P*. *rapae*, and provides insight into the mechanism of the *P*. *puparum* venom/*P*. *rapae* immune response interaction. The nature of the venom component that causes this effect is unknown, and *P*. *puparum* venom is likely to be a complex mixture with many components. Characterisation of parasitoid venom components will allow assays to identify which components interfere with host immune signalling transduction, and exploration of targets for their activity in the host.

## Materials and Methods

### Insect Rearing

Cultures of *P*. *rapae* and *P*. *puparum* were maintained as described previously and used in all experiments [32]. After emerging, *P*. *puparum* females were collected and held in glass containers, fed *ad lib* on 20% (v/v) honey solution to lengthen life span for 3-4 days until dissection of the venom reservoir and gland.

### Crude Venom Preparation

Venom collection was described by Wu et al [31]. Five hundred glands and reservoirs were then transferred to a sterilized 1.5 ml Eppendorf tube and centrifuged at 12,000 g for 20 min at 4°C. The supernatant was collected and then filtered through a 0.22 µm cellulose acetate filter. The crude venom solution was diluted with PBS to the final concentration of 2 venom reservoir equivalents (VREs)/µl immediately before use.

### Full length cDNA cloning

An immunologically naive pupa was injected with approximately 50 Sephadex A-50 beads (GE healthcare, New Jersey, USA) by 801 RN micro-syringe (Hamilton Bonaduz AG, Bonaduz, Switzerland) to stimulate its immune responses. Beads were prepared for injection as previously described [32]. At 4 h post immune induction, the total RNA sample was isolated using Trizol Reagent (Invitrogen, California, USA) from the pupa. First-strand cDNA was synthesized using a SMART ™ RACE cDNA Amplification Kit (Clontech, California, USA), following the instructions for this kit. The sequence of the *Pr-CTL* cDNA fragment previously identified [32] was used to predict a gene-specific primer (5′-CTTCAAGGTGACATCTTAGCCGAG-3′) for 5′-RACE. The thermal cycling conditions for 5′-RACE were 32 cycles of 94°C, 30 s; 60°C, 30 s; and 72°C, 30 s followed by incubation at 72°C, 10 min. PCR products were separated by electrophoresis on a 1.0% agarose gel. The purified PCR products were cloned into pGEM ® T-Easy Vector (Promega, Wisconsin, USA) and then sequenced.

### Sequence analysis

DNA Star software package (Version 5.02) was used to assemble the cDNA fragment sequence and to find the ORF of full length cDNA. Signal peptide was predicted by SignalP software (Version 3) (http://www.cbs.dtu.dk/services/SignalP/). Sequence comparison and phylogenetic analysis were performed by MEGA (Version 4) software [59]. Sequences were aligned using Clustal W2 (http://www.ebi.ac.uk/Tools/clustalw2/index.html). A phylogenetic tree was constructed by the unweighted pair group method with arithmetic mean (UPGMA), with statistical analysis by bootstrap method using 1,000 repetitions. The sequences used for the analyses are listed in Table S1.

### Gene expression profile analysis

The effects of different treatments on immune induction and suppression were carried out on immunologically naive pupae of the host *P*. *rapae* (1 d after pupation). Pupae were exposed to parasitoid females to obtain parasitized hosts. In other experiments, pupae were injected with 5×10^4^ pfu of *M*. *luteus* (Molecular Probe, Eugene, USA) and *E*. *coli* K12, 50 Sephadex beads, and beads + venom (2VREs) suspended in 1 µl of sterilized PBS, respectively. PBS injected pupa was set as the control. Each treatment or control was repeated 5 times. Total RNA samples were isolated from the pupae of each treatment and control, using Trizol Reagent, at 1 h post treatment. Total RNA samples were treated with TURBO ™ DNase (Ambion, Texas, USA) to remove DNA contaminants. First-strand cDNA was synthesized using SuperScript ™ III First-Strand Synthesis System (Invitrogen) and random hexamers as primer. Each 10 µl of first-strand cDNA product was diluted with 190 µl of sterilized water before utilization. The rq-rtPCR experiments were carried out, using *P*. *rapae 18S rRNA* gene as internal control. Primer pairs of *Pr-CTL* and *18S rRNA* genes were designed using Primer3 (http://frodo.wi.mit.edu/cgi-bin/primer3/) and listed in Table S2. Each 25 µl reaction contained 12.5 µl iQ TM SYBR® Green Supermix (Bio-Rad, New Jersey, USA), 1 µl forward primer (200 nM), 1 µl reverse primer (200 nM) and 10.5 µl diluted cDNA. The thermal cycling conditions were 95°C, 30 s; and 40 cycles of 95 °C, 5 s; 51°C, 20 s; and 72°C for 20 s. Amplification was monitored on iCycler iQ™ Real-Time PCR Detection System (Bio-Rad). The specificity of the SYBR-Green PCR signal was further confirmed by melting curve analysis. The experiments were repeated 5 times. The mRNA expression was quantified using the comparative cross-threshold method [60].

For the time course assay of changes in *Pr-CTL* expression in response to immune challenge, total RNA samples were isolated from the treated host pupae at 0, 1, 4, 8, 12 and 24 h, respectively, post beads injection. The experiments were repeated 5 times. The mRNA transcript levels of *Pr-CTL* were quantified using rq-rtPCR, as described above.

For the time course assay of venom effects on *Pr-CTL* expression, 50 Sephadex beads + venom (2 VREs) were injected into an immunologically naive pupa. To assay the dose effect, beads + different doses of venom, including 0, 0.5, 1 and 2 VREs, were respectively injected into an immunologically naive pupa. Total RNA samples were isolated from the injected pupae, at 1, 4, 8, 24 and 48 h post treatments for the time course, and 8 h for the dose effect assays. The transcript level of the *Pr-CTL* gene in each total RNA sample was estimated as described above.

To assess expression of *Pr-CTL* in different tissues, the treated host pupae were sampled, and the hemolymph was collected at 1 h after injection of beads as previously described [32]. After hemolymph collection, cuticle, gut, and fat body were dissected. Plasmatocytes and granulocytes were separated, using a modification of the method of Wiesner and Götz [61]. Briefly, 1 cm^3^ of loose nylon wool fiber (Wako, Tokyo, Japan) was inserted into a 5 ml sterilized syringe to plug the outlet. The inner wall of the syringe and the nylon wool fiber were washed repeatedly with anticoagulant solution, then the syringe containing nylon wool fiber was vertically fixed on a steel frame and the outlet was sealed from the outside using Parafilm membrane. One milliliter of hemolymph was slowly added into the fixed syringe. The hemolymph and the nylon wool fiber were co-incubated for 1 h at 28°C in order to let the granulocytes adhere to the fiber firmly. The syringe outlet was then opened, and 10 ml of anticoagulant solution was poured through the syringe, collecting the eluted liquid. Most of granulocytes were adsorbed on fiber, while most of plasmatocytes were in the eluted solution, which was centrifuged at 200 g for 10 min at 8°C to prepare the plasmatocytes. In *P*. *rapae*, 97% of hemocytes were plasmatocytes or granulocytes, and this method separated these two cell types in sufficient purity for subsequent experiments. Total RNA samples were isolated from all tissues collected as described above. The experiments were repeated 5 times. The mRNA transcript levels of *Pr-CTL* were quantified using rq-rtPCR, as described previously.

### Production of antibody against Pr-CTL

The cDNA fragment encoding the mature peptide of Pr-CTL was sub-cloned into PET 28a vector (Novagen, New Jersey, USA), using specific primers Pr-CTL-subSP containing a SacI site at 5′-end and Pr-CTL-subAP containing a SalI site at 5′-end, which were listed in Table S2. PCR was performed as following: 95°C, 2 min; and 30 cycles of 95°C, 30 s; 59°C, 30 s; and 72°C, 1 min followed by 72°C, 10 min. The PCR product was digested with SacI and SalI enzymes respectively. The product was inserted into SacI/SalI digested PET 28a vector and then transformed into *E*. *coli* BL21 (DE3) competent cells (Takara, Tokyo, Japan). Expression of the recombinant was induced by IPTG (0.5 mM), and detected by immunoblotting using anti-His-tag monoclonal antiserum. The recombinant protein was purified under denaturing conditions in 8 M urea using nickel-nitrilotriacetic acid resin according to the manufacturer's instructions (Qiagen, Hilden, Germany). Recombinant protein (3 mg) was separated by 12% SDS-PAGE under reducing conditions, and the gel slice containing recombinant Pr-CTL was cut out and used as an antigen to inject rabbits for polyclonal antibody preparation.

### Immunoblotting analysis

Total protein samples were extracted from the treated pupae, dissected tissues and cell-free plasma mentioned above. Briefly, both pupae and different tissues were first homogenized in liquid N_2_ and the samples were then homogenized again using PBS. The extractions and hemolymph samples collected from treated pupae were centrifuged at 10,000 g for 20 min at 4°C. The supernatants were collected as total protein preparations. Protein quantification was determined by the Bradford method [62]. Each sample (20 µg) was separated by 12% SDS-PAGE under reducing conditions. Immunoblotting analysis was performed using rabbit polyclonal antiserum against Pr-CTL as the primary antibody (diluted 1∶2500) and goat anti-rabbit IgG-horseradish peroxidase conjugate (Sigma-Aldrich, Taufkirchen, Germany; diluted 1∶5000) as the secondary antibody.

### Immunolocalization of Pr-CTL in hemocytes

Hemolymph was collected from one *P*. *rapae* pupa injected with only 5-10 Sephadex beads as described previously, at 0, 4, 8 h, respectively, post treatment. Then, 30 µl of samples of hemolymph were mixed with 170 µl TC-100 medium (Sigma-Aldrich) containing 50 µg/ml tetracycline and 2 µl saturated 2-phenylthiourea (PTU). The diluted hemolymph was added to each well of a 12-well multi-test slide (ICN Biomedicals, California, USA) and the hemocytes adhered to the slide for 15 min at in moist chamber at 25°C to form a monolayer, which did not encapsulate to the surface of injected beads. Plasma was removed from the monolayer, which was then washed with PBS. The monolayer was fixed and blocked as described previously [6]. Rabbit anti-Pr-CTL polyclonal antiserum diluted in 0.5% BSA at 1∶400 was added to each well and incubated with the monolayer for 1 h. After removing the antiserum solution, the monolayer was washed with PBS for 3 times, each for 5 min. Then 100 µl of goat anti- rabbit IgG-fluorescein isothiocyanate (FITC) conjugate (Sigma-Aldrich) diluted at 1∶200 was used as the secondary antibody and incubated with the monolayer for 45 min. The monolayer was washed again with PBS for 3 times, and at the last washing, the nuclei of hemocytes were stained with 4′, 6-diamidino-2-phenylindole (DAPI) at 0.5 µg/ml. Hemocytes were observed by confocal microscope (Zeiss, New York, USA).

In addition, hemolymph was collected from *P*. *rapae* pupae (one per treatment) injected with PBS, 5-10 beads, beads + venom (2VREs), respectively, at 4 h post injection. Then, the immunolocalization experiment was performed as described above.

### Down-regulation of *Pr-CTL* expression by RNA Interference

The dsRNA samples containing coding sequences for Pr-CTL and green fluorescent protein (EGFP, control) were synthesized *in vitro*, using T7 RiboMAX ™ Express RNAi System (Promega), according to its instruction. Briefly, pairs of primers were designed for *Pr-CTL* and *EGFP* genes (listed in Table S2). DNA fragments for production of sense single-strand RNA (ssRNA) and anti-sense ssRNA were amplified from plasmid templates containing the *Pr-CTL* gene sequence (in the pGEM ® T-Easy Vector) and the *EGFP* gene sequence (pEGFP vector; Clontech) by PCR. For sense ssRNA from *Pr-CTL* gene, the primer pair T7*PrCTL*-F and *PrCTL*-R were used, and for its anti-sense ssRNA, *PrCTL*-F and T7*PrCTL*-R were used. Similar primer pairs were used to amplify fragments for *EGFP* ssRNAs. The thermal cycling conditions for PCR were 95°C, 3 min; and 30 cycles of 95°C, 30 s; 54°C, 30 s; and 72°C, 30 s; followed by 72°C, 10 min. Using the PCR products as DNA templates, *in vitro* transcription of ssRNA was carried out following the kit instruction. Sense and anti-sense ssRNA samples were annealed to form the dsRNA. After annealing, the dsRNA solution was treated with RNase A (Takara) and DNase (Ambion) at 37°C for 30 min to digest un-annealed ssRNA and DNA template. Finally, treated dsRNA was purified by ethanol precipitation, according to the kit instructions. The integrity of dsRNA was confirmed by gel electrophoresis. dsRNA quantity was determined spectrophotometrically (using A _260/280_).

Immunologically naive pupae (1 d after pupation) were injected with 20 µg of dsRNA from *Pr-CTL* and *EGFP* genes suspended in 2 µl of water, respectively. PBS injection was set as the control. Each treatment or control was replicated 48 times, 5 times for rq-rtPCR, 3 times for immunoblotting, 5 times for antimicrobial, 5 times for phagocytosis and 25 times for encapsulation assays.

For rq-rtPCR, the treated pupae were first co-injected with 50 Sephadex beads at 12 h post dsRNA (or PBS) injection. At 4 h post beads challenge, total RNA sample of each treated or control pupa was isolated, and then the transcripts levels of *Pr-CTL*, two antimicrobial peptide genes including *Pr-cecA* and *lys* [32], two proPO activating protein (*Pr-PAP1* and *Pr-PAP3*) genes, a scavenger receptor (*Pr-SR*) gene, and a glutathione s-transferase (*Pr-GST*) gene were analyzed. *Pr-GST* gene was used as a control. Beside *Pr-CTL* gene, the full length cDNAs or the cDNA fragments of 6 genes mentioned above have been cloned previously in our lab. The parameters for rq-rtPCR and the primer pairs for the *Pr-CTL* gene were described previously. Parameters and primer pairs for *Pr-cecA, Pr-lys, Pr-PAP1, Pr-PAP3*, *Pr-SR* and *Pr-GST* genes were based on cDNA sequences cloned previously (results not presented), and are shown in Text S1. Total protein of each treated or control pupa was also extracted at 4 h post beads challenge, and the level of Pr-CTL was estimated by immunoblotting performed as described above.

### Phagocytosis assay

Thirty microliters of the collected hemolymph from pupae at 12 post dsRNA (or PBS) injection was diluted with 470 µl anticoagulant solution (0.9% NaCl, 0.942% KCl, 0.082% CaCl_2_, 2% EDTA). Diluted hemolymph was centrifuged at 200 g for 10 min at 8°C, and the hemocyte pellet was resuspended in PBS. 100 µl of hemocyte suspension was added to a cover slide treated with Poly-L-Lysine (Sigma-Aldrich). The slide was left in moist chamber for 20 min at 25°C to allow a hemocyte monolayer to form. The monolayer was washed briefly with PBS, and overlaid with 50 µl suspension of FITC labeled *E*. *coli* K12 (Molecular Probes), to give a ratio of hemocytes/*E*. *coli* of 1/10. The monolayer and *E*. *coli* were co-incubated for 1 h in darkness. The monolayer was put into a cell culture dish (Corning, New York, USA), and washed carefully with 2 ml of PBS. The monolayer was overlaid with 0.5% trypan blue dye solution for 10 min, in order to quench the non-phagocytosed *E*. *coli*, then fixed using 1.5% glutaraldehyde and observed at 400× under FITC and differential interference contrast (DIC) modes using a TE2000-U fluorescence microscope (Nikon, Tokyo, Japan). The percentage of hemocytes phagocytosing FITC-labeled *E*. *coli* was estimated from 5 random microscopic fields by counting at least 200 hemocytes in each monolayer, and used to calculate the phagocytosis rate.

### Encapsulation assays

A 96-well cell culture plate (BD Falcon, California, USA) was first treated with 5% bovine serum albumin (BSA) at 4°C over night. Thirty microliters of hemolymph were collected from the pupae at 12 h post dsRNA (or PBS) injection, and then was combined with 168 µl TC-100 medium (Sigma-Aldrich), containing 50 µg/ml tetracycline and 50 µg/ml ampicillin. The diluted hemolymph was immediately added to each well of the BSA-coated plate. Hemocytes were allowed to settle for 15 min. Then, 20 Sephadex beads suspended in 2 µl of TC-100 medium was added to each well, and the plate was incubated at 25°C. The encapsulated Sephadex beads were observed after 4 h incubation, under a phase contrast microscope (Leica, Wetzlar, Germany) at 400×, and the percentage of encapsulated beads per 100 beads deduced from observation of 5 wells was calculated. The encapsulation assay was repeated 5 times.

For the time course assay of venom effects on encapsulation, hemocytes were directly collected from immunologically naive pupae as described, and diluted hemolymph solutions were prepared and treated with venom (2 VREs). Encapsulation was assessed *in vitro* at 1, 4, 8, 24 and 48 h post-treatment as described above. The experiments were repeated 5 times. To assess a potential dose effect, beads + venom doses of 0, 0.5, 1 and 2 VREs, were added into immunologically naive and diluted hemolymph solutions. Hemocyte encapsulation assays were performed *in vitro* as described above, for each treatment at 8 h post beads + venom appending. The experiments were repeated 5 times.

### Antimicrobial assay

For the assay, the dsRNA (or PBS) injected pupae were injected with 50 beads at 12 h post dsRNA or PBS treatments. At 4 h post beads challenge, the hemolymph was collected from the induced pupae, and then was centrifuged at 200 g for 10 min at 8°C. The antimicrobial activities of 10 µl undiluted hemolymph supernatant samples were determined. Antimicrobial activity was analyzed by the inhibition zone method [63], [64]. In brief, 12 ml of melted agar in Luria-Bertani medium pH 7.2, containing logarithmic phase cells of *E*. *coli* K12 were added into the sterilized Petri dishes. The plates were incubated for 24 h at 37°C, and the inhibition zone squares were recorded, after subtraction of the well diameter. 10 µl aliquots of ampicillin at different concentrations were used as the positive control.

### PO Activity Assay

Hemolymph of each treated pupa was collected at 12 h post dsRNA (or PBS) injection, then directly added into Tris-Ca^2+^ buffer (100 mM Tris-HCl,100 mM NaCl,1 mM CaCl_2_,pH 7.5), containing 50 Sephadex beads. After beads challenge, the mixture was collected and centrifuged at 10,000 g for 20 min at 4°C. The supernatant was taken as a crude enzyme preparation. Protein was quantified by the Bradford method using BSA to create a standard curve. For determination of PO activity, a 1 ml mixture containing 2 mM dopamine (Sigma-Aldrich), 50 mM sodium phosphate buffer (pH 6.0) and 10 mg of crude enzyme protein was incubated at 28°C and the increase in absorbance at 490 nm was continuously monitored. One unit of enzyme activity was defined as an increase of 0.001 in absorbance/minute/mg protein.

### Immunolocalization of recombiant Pr-CTL in parasitoid egg

Pr-CTL was recombinantly expressed and purified as the description above. Purified Pr-CTL was diluted to a final concentration of 0.02 mg/ml and renatured by three-step dialysis as reported previously [6]. The concentration of renatured protein was determined by the Bradford method using BSA to create a standard curve.

The mature eggs of *P*. *puparum* were sampled by dissecting ovaries of female wasps 72 h after mating, as the previous description [65]. To test binding of recombinant Pr-CTL to the eggs of *P*. *puparum*, renatured protein was diluted 200 µg/ml with PBS containing 0.5% BSA. Two hundred microliters of the protein solution was added to each well of a 12-well multi-test slide and incubated with eggs for 2 h at room temperature. Then the protein solution was discarded and the eggs were washed, fixed and blocked as described above. Mouse anti-His-tag monoclonal antiserum (Sigma-Aldrich) diluted in PBS containing 0.5% BSA at 1∶400 was used as the primary antibody. Goat anti-mouse IgG-FITC conjugate (Sigma-Aldrich) was used as the secondary antibody, diluted in PBS containing 0.5% BSA at 1∶200. One hundred microliters of the primary and secondary antibody solutions were added to each well sequentially and incubated at room temperature for 1 h and 45 min, respectively. The eggs were washed again with PBS for 3 times, and at the last washing, the nuclei of cells on the surface of the eggs were stained with DAPI at 0.5 µg/ml. Recombinant Pr-HSP 70 also fused with His-tag at its N-terminus (expressed by our lab previously), a heat shock protein of host *P*. *rapae*, was used as a control protein. The eggs were observed by confocal microscope.

### Statistical Analysis

The significance of differences between parameters measured for different treatments was estimated using analysis of variance (ANOVA). Means were compared using LSD tests, and statistical significance was set at *P*<0.05. Partial percentages data were first transformed to a quasi-normal distribution by arcsine-square root values prior to the statistical analysis. All analyses were performed by DPS software (version 8.01) [66].

## Supporting Information

Figure S1 Nucleotide and deduced amino acid sequences of *Pr-CTL*. *Pr-CTL* cDNA (above) and amino acid (below) sequences are shown with a predicted signal peptide, and cleavage site is indicated by a black triangle. Two potential N-linked glycosylation sites are marked with pink circles. The first and second CRDs are indicated by red and blue square brackets, respectively. The EPD and EPN motifs are shaded in sage green color. A polyadenylation signal sequence is double underlined.
(TIF)

Figure S2 Phylogenetic analysis between Pr-CTL and other insect C-type lectins. Construction was performed on the basis of the homology sequences calculated from the complete amino acid sequences of CTLs, using UPGMA method. Sequences were selected from NCBI databases. The sequence of galactose binding protein 4 of *Caenorhabditis elegans* (Ce-GBP 4) is used as the out-group. The sequences used were listed in the Table S2. Pr-CTL was boxed.
(TIF)

Figure S3 Analysis of recombinant Pr-CTL by SDS-PAGE and immunoblotting. The total proteins of the non-induced *E*. *coli* BL21 (DE3) clone containing recombinant plasmid (20 µg for coomassie blue staining, line “N” in panel of SDS-PAGE), and the total proteins of the induced clone (20 µg, line “I” in panel of SDS-PAGE) were analyzed by 12% SDS-PAGE under reducing condition. After SDS-PAGE, the recombinantly His-tag fused Pr-CTL expressed by induced the clone was detected by immunoblotting, using anti-His-tag monoclonal antiserum as the first antibody. The asterisks represent the recombinant protein both in SDS-PAGE and immunoblotting.
(TIF)

Table S1 Sequences used in multiple alignments and phylogenetic tree construction for *Pr-CTL*, including galactose binding protein 4 of *Caenorhabditis elegans* (Ce-GBP 4) as out-group sequence.
(DOC)

Table S2 Primers used in this research article are presented.
(DOC)

Text S1 Parameters of rq-rtPCR and pairs of primers for *Pr-cecA*, *Pr-lys*, *Pr-PAP1*, *Pr-PAP3*, *Pr-SR*, and *Pr-GST* genes of *P*. *rapae* cloned in our lab previously, are presented.
(DOC)
