# Supplementary figures and images for: Venom of Parasitoid, Pteromalus puparum, Suppresses Host, Pieris rapae, Immune Promotion by Decreasing Host C-Type Lectin Gene Expression
Source: PLoS One. 2011 Oct 26;6(10):e26888. doi: 10.1371/journal.pone.0026888 (PMC3202585; doi:10.1371/journal.pone.0026888)

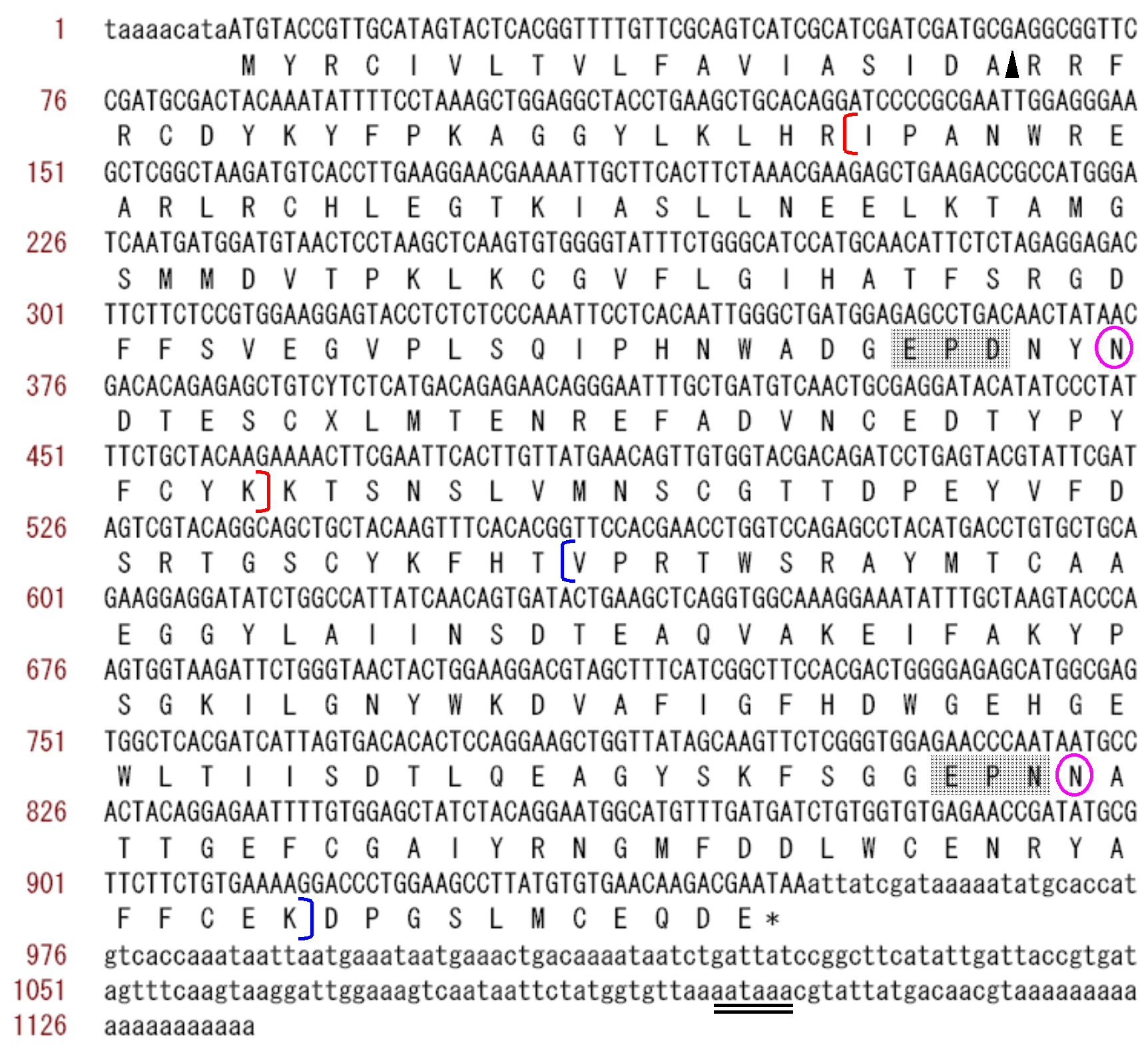

Supplement: Figure S1 — Nucleotide and deduced amino acid sequences of Pr-CTL. Pr-CTL cDNA (above) and amino acid (below) sequences are shown with a predicted signal peptide, and cleavage site is indicated by a black triangle. Two potential N-linked glycosylation sites are marked with pink circles. The first and second CRDs are indicated by red and blue square brackets, respectively. The EPD and EPN motifs are shaded in sage green color. A polyadenylation signal sequence is double underlined. (TIF) [file pone.0026888.s001.tif]

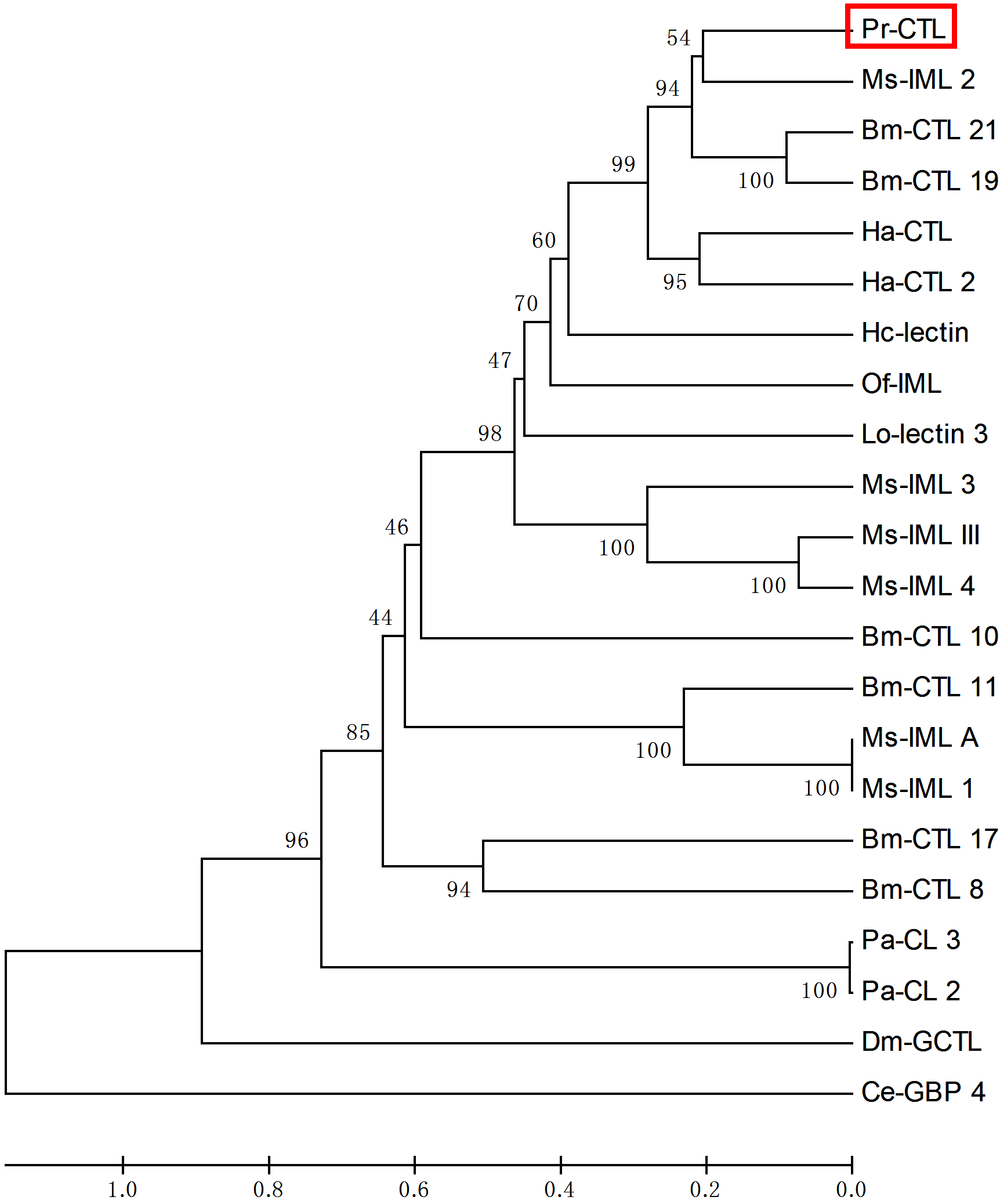

Supplement: Figure S2 — Phylogenetic analysis between Pr-CTL and other insect C-type lectins. Construction was performed on the basis of the homology sequences calculated from the complete amino acid sequences of CTLs, using UPGMA method. Sequences were selected from NCBI databases. The sequence of galactose binding protein 4 of Caenorhabditis elegans (Ce-GBP 4) is used as the out-group. The sequences used were listed in the Table S2. Pr-CTL was boxed. (TIF) [file pone.0026888.s002.tif]

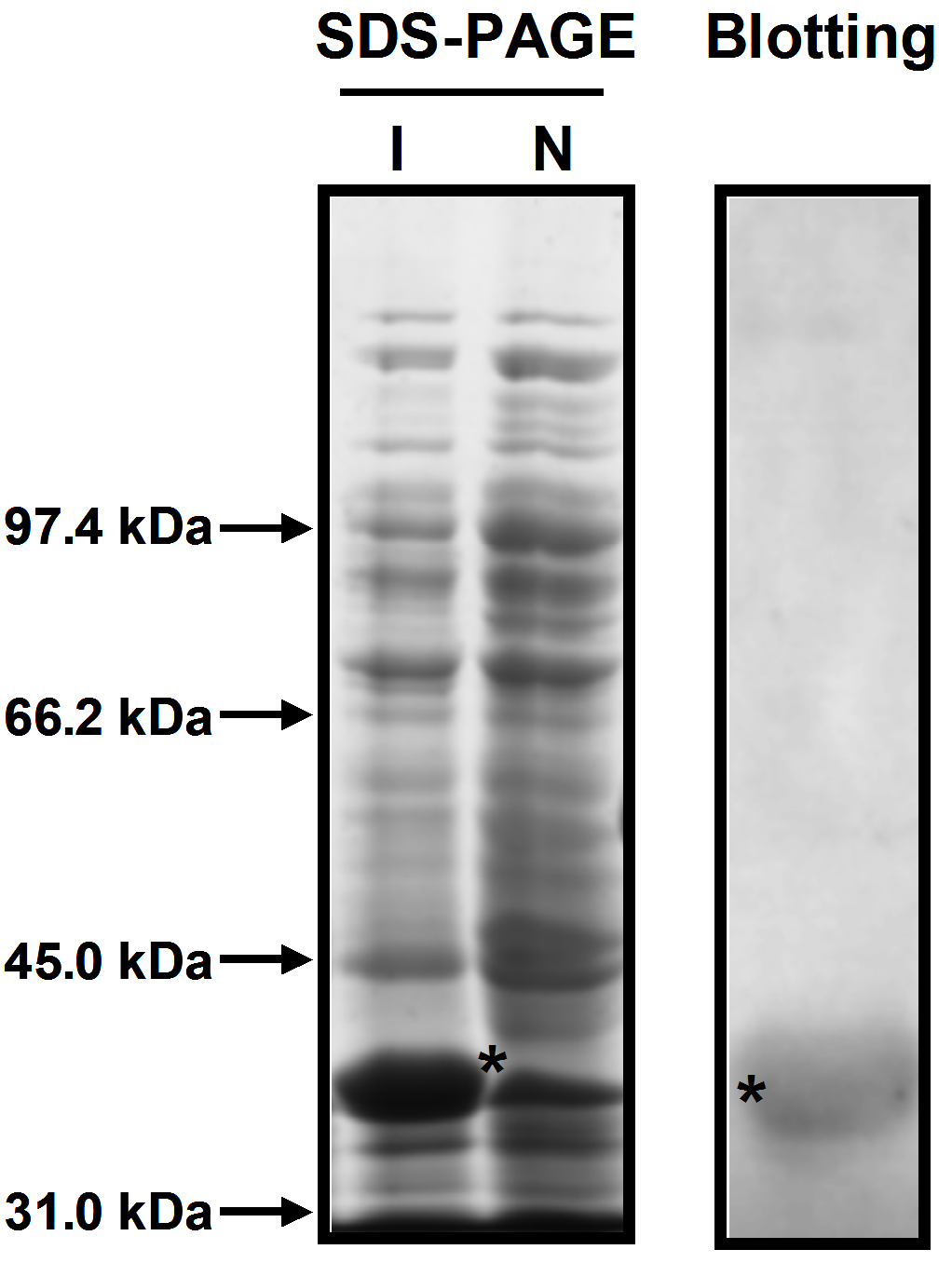

Supplement: Figure S3 — Analysis of recombinant Pr-CTL by SDS-PAGE and immunoblotting. The total proteins of the non-induced E. coli BL21 (DE3) clone containing recombinant plasmid (20 µg for coomassie blue staining, line “N” in panel of SDS-PAGE), and the total proteins of the induced clone (20 µg, line “I” in panel of SDS-PAGE) were analyzed by 12% SDS-PAGE under reducing condition. After SDS-PAGE, the recombinantly His-tag fused Pr-CTL expressed by induced the clone was detected by immunoblotting, using anti-His-tag monoclonal antiserum as the first antibody. The asterisks represent the recombinant protein both in SDS-PAGE and immunoblotting. (TIF) [file pone.0026888.s003.tif]
