# Supplementary material for: Venom of Parasitoid, Pteromalus puparum, Suppresses Host, Pieris rapae, Immune Promotion by Decreasing Host C-Type Lectin Gene Expression
Source: PLoS One. 2011 Oct 26;6(10):e26888. doi: 10.1371/journal.pone.0026888 (PMC3202585; doi:10.1371/journal.pone.0026888)
Supplement: Table S1 — Sequences used in multiple alignments and phylogenetic tree construction for Pr-CTL, including galactose binding protein 4 of Caenorhabditis elegans (Ce-GBP 4) as out-group sequence. (DOC) [file pone.0026888.s004.doc]

Table S1. Sequences used in multiple alignments and phylogenetic tree construction.

| **Name** | **Accession No.** | **Species** |
| --- | --- | --- |
| Pr-CTL | - | *Pieris rapae* |
| Ms-IML 2 | AAC33576 | *Manduca sexta* |
| Bm-CTL 21 | NP_001037056 | *Bombyx mori* |
| Bm-CTL 19 | NP_001165396 | *Bombyx mori* |
| Ha-CTL | ABF83203 | *Helicoverpa armigera* |
| Ha-CTL 2 | ACI32834 | *Helicoverpa armigera* |
| Hc-lectin | AAD09286 | *Hyphantria cunea* |
| Of-IML | ABZ81710 | *Ostrinia furnacalis* |
| Lo-lectin 3 | AAV91450 | *Lonomia obliqua* |
| Ms-IML 3 | AAV41236 | *Manduca sexta* |
| Ms-IML III | CAL25134 | *Manduca sexta* |
| Bm-CTL 8 | NP_001124372 | *Bombyx mori* |
| Pa-CL 3 | BAA82266 | *Periplaneta americana* |
| Pa-CL 2 | BAA82267 | *Periplaneta americana* |
| Dm-GCTL | AAF53793 | *Drosophila melanogaster* |
| Ms-IML 4 | AAV41237 | *Manduca sexta* |
| Bm-CTL 10 | NP_001091784 | *Bombyx mori* |
| Bm-CTL 11 | NP_001037076 | *Bombyx mori* |
| Ms-IML A | AAC33576 | *Manduca sexta* |
| Ms-IML 1 | ADD13530 | *Manduca sexta* |
| Ce-GBP 4 ^a^ | NP_497763 | *Caenorhabditis elegans* |

^a^ This sequence is used as the out-group.
