# Supplementary material for: Venom of Parasitoid, Pteromalus puparum, Suppresses Host, Pieris rapae, Immune Promotion by Decreasing Host C-Type Lectin Gene Expression
Source: PLoS One. 2011 Oct 26;6(10):e26888. doi: 10.1371/journal.pone.0026888 (PMC3202585; doi:10.1371/journal.pone.0026888)
Supplement: Table S2 — Primers used in this research article are presented. (DOC) [file pone.0026888.s005.doc]

Table S2. Primers are used in this research article.

| **Application** | **Primer name** | **Primer Sequences (from 5' to 3')** |
| --- | --- | --- |
| rq-rtPCR | Pr-CTL-rtSP | TCCAGAGCCTACATGACCT |
|  | Pr-CTL-rtAP | GTTACCCAGAATCTTACCACTT |
| Internal control | Pr-18S-rtSP | TTTGCCTTATCAACTTTCG |
|  | Pr-18S-rtAP | TGTGGTAGCCGTTTCTCA |
| Sub-cloning | Pr-CTL-subSP ^a^ | *TA*GAGCTC TAGAGCTCAGGCGGTTCCGATGCGAC |
|  | Pr-CTL-subAP ^a^ | *AT*GTCGAC ATGTCGACTTATTCGTCTTGTTCACACATAAGGC |
| dsRNA synthesis | T7PrCTL-F ^b^ | *GGATCG*TAATACGACTCACTATAGGAGAGCCTGACAACTATAACGAC |
|  | PrCTL-F ^b^ | AGAGCCTGACAACTATAACGAC |
|  | T7GFP-F ^b^ | *GGATCG*TAATACGACTCACTATAGGAAGGGCGAGGAGCTGTTCACCG |
|  | GFP-F ^b^ | AAGGGCGAGGAGCTGTTCACCG |
|  | T7PrCTL-R ^b^ | *GGATCG*TAATACGACTCACTATAGGGTTACCCAGAATCTTACCACTT |
|  | PrCTL-R ^b^ | GTTACCCAGAATCTTACCACTT |
|  | T7GFP-R ^b^ | *GGATCG*TAATACGACTCACTATAGGCAGCAGGACCATGTGATCGCGC |
|  | GFP-R ^b^ | CAGCAGGACCATGTGATCGCGC |

^a^ Extra bases upstream of the restriction site (underline) are the protective bases.

^b^ Extra bases (italic letters) upstream of the minimal T7 RNA polymerase promoter (underline) sequence are able to increase yield by allowing more efficient polymerase binding and initiation.
