# Supplementary material for: Venom of Parasitoid, Pteromalus puparum, Suppresses Host, Pieris rapae, Immune Promotion by Decreasing Host C-Type Lectin Gene Expression
Source: PLoS One. 2011 Oct 26;6(10):e26888. doi: 10.1371/journal.pone.0026888 (PMC3202585; doi:10.1371/journal.pone.0026888)
Supplement: Text S1 — Parameters of rq-rtPCR and pairs of primers for Pr-cecA, Pr-lys, Pr-PAP1, Pr-PAP3, Pr-SR, and Pr-GST genes of P. rapae cloned in our lab previously, are presented. (DOC) [file pone.0026888.s006.doc]

**Supporting methods.**

The thermal cycling condition of rq-rtPCR for *Pr-cecA*, *Pr-lys*, *Pr-PAP1*, *Pr-PAP3* and *Pr-SR* genes were all 95 ^o^C, 30 s; and 40 cycles of 95 ^o^C, 5 s; 51 ^o^C, 20 s; and 72 ^o^C for 20 s. The thermal cycling condition for *Pr-GST* was 95 ^o^C, 30 s; and 40 cycles of 95 ^o^C, 5 s; 57 ^o^C, 20 s; and 72 ^o^C for 20 s. Amplification was monitored on iCycler iQ ^TM^ Real-Time PCR Detection System (Bio-Rad). The specificity of the SYBR-Green PCR signal was further confirmed by melting curve analysis. The mRNA expression was quantified using comparative CT method, using *18S rRNA* gene as internal control. Its thermal cycling condition and Primers design has been reported by Fang et al [32]. Primers for *Pr-cecA* were Pr-cecA-SP (5’- TTTCGCAACCACCTACAT-3’) as well as Pr-cecA-AP (5’-TTCCAGCATTTCCATCAG-3’), for *Pr-lys* were Pr-lys-SP (5’- TTGGGTATGTCTCGTTGAA -3’) as well as Pr-lys-AP (5’- TTGTGATGTCGTCCGTTGT -3’), for *Pr-PAP1* were Pr-PAP1-SP (5’- CTGCCAGACTACCAACAAC -3’) as well as Pr-PAP1-AP (5’- AGATTCCACCAGCACAAAG -3’), for *Pr-PAP3* were Pr-PAP3-SP (5’- GTTTCACTGGACGGGACT -3’) as well as Pr-PAP3-AP (5’- GTTTAGCGGAAGGGTTGT -3’), for *Pr-SR* were Pr-SR-SP (5’- CTCACTGATGTCTCGGAAAG -3’) as well as Pr-SR-AP (5’- AGAGGAGGACTGGGTGTAAT -3’), and for *Pr-GST* were Pr-GST-SP (5’- TTCCTTCCATACGGTCAACTGC -3’) as well as Pr-GST-AP (5’- TTCCCAGGCGTCAGTAGGTAGA -3’).
